# Choking

**Published:** 1886-05

**Authors:** Jerome Walker


### Choking.

A baby or young child may hold its breath while there is food in the mouth, simply because it can not obtain more food or can not have its own way. As soon as the spasm of the muscles of the throat relaxes, an inspiration occurs, air is forcibly drawn into the lungs, and if particles of food have not already been removed from the mouth and throat by one's finger, they are likely to block up the larynx and cause suffocation. In other words, they are "foreign bodies." Children just passing out of babyhood who are allowed to feed themselves at table and to eat whatever they want, run great risks of suffocation by large mouthfuls of food. No careful parent who has repeatedly observed a baby's manner of cramming the mouth full and of gulping food, if left to himself, doubts that suffocation may thereby be caused. To reduce the danger to the minimum, therefore, additional food should not be given until the baby's mouth is quite empty, and the mother should not intrust the feeding to other hands then her own, unless, indeed, she intelligently supervises it.—*Dr. Jerome Walker*.

